# Potential of trans fats policies to reduce socioeconomic inequalities in mortality from coronary heart disease in England: cost effectiveness modelling study

**DOI:** 10.1136/bmj.h4583

**Published:** 2015-09-15

**Authors:** Kirk Allen, Jonathan Pearson-Stuttard, William Hooton, Peter Diggle, Simon Capewell, Martin O’Flaherty

**Affiliations:** 1 Lancaster Medical School, Lancaster University, Lancaster LA1 4YW, UK; 2 Department of Public Health and Policy, Liverpool University, Liverpool L69 3GB, UK; 3 Division of Medical Sciences, University of Oxford, Oxford OX3 9DU, UK; 4 Pembroke College Alumni, University of Oxford, Oxford OX1 1DW, UK

## Abstract

**Objectives** To determine health and equity benefits and cost effectiveness of policies to reduce or eliminate trans fatty acids from processed foods, compared with consumption remaining at most recent levels in England.

**Design** Epidemiological modelling study.

**Setting** Data from National Diet and Nutrition Survey, Low Income Diet and Nutrition Survey, Office of National Statistics, and health economic data from other published studies

**Participants** Adults aged ≥25, stratified by fifths of socioeconomic circumstance.

**Interventions** Total ban on trans fatty acids in processed foods; improved labelling of trans fatty acids; bans on trans fatty acids in restaurants and takeaways.

**Main outcome measures** Deaths from coronary heart disease prevented or postponed; life years gained; quality adjusted life years gained. Policy costs to government and industry; policy savings from reductions in direct healthcare, informal care, and productivity loss.

**Results** A total ban on trans fatty acids in processed foods might prevent or postpone about 7200 deaths (2.6%) from coronary heart disease from 2015-20 and reduce inequality in mortality from coronary heart disease by about 3000 deaths (15%). Policies to improve labelling or simply remove trans fatty acids from restaurants/fast food could save between 1800 (0.7%) and 3500 (1.3%) deaths from coronary heart disease and reduce inequalities by 600 (3%) to 1500 (7%) deaths, thus making them at best half as effective. A total ban would have the greatest net cost savings of about £265m (€361m, $415m) excluding reformulation costs, or £64m if substantial reformulation costs are incurred outside the normal cycle.

**Conclusions** A regulatory policy to eliminate trans fatty acids from processed foods in England would be the most effective and equitable policy option. Intermediate policies would also be beneficial. Simply continuing to rely on industry to voluntary reformulate products, however, could have negative health and economic outcomes.

## Introduction

Cardiovascular diseases are the most common cause of mortality in the United Kingdom, with around 180 000 deaths in 2010 and economic costs of £19bn (€26bn, $30bn) in 2009.^1^ Coronary heart disease is the most common type of cardiovascular disease, causing some 80 000 deaths in 2010. Premature mortality from coronary heart disease is substantially higher among the most disadvantaged socioeconomic groups,^2^ and this inequality might increase in the future.^3^

Trans fatty acids are unsaturated fatty acids with at least one double bond in a *trans* position. They are produced naturally in small quantities in the stomachs of ruminants (such as cows and sheep) but are more commonly found in the diet from partially hydrogenated vegetable oils in processed foods. Trans fatty acids are desirable to the food industry because they allow longer shelf life, thermodynamic stability, and enhanced palatability. They increase low density lipoprotein cholesterol concentrations, decrease high density lipoprotein cholesterol concentrations, and can also cause systemic inflammation as well as endothelial dysfunction.^4^ A meta-analysis of pooled prospective studies found that for every 2% of total energy (%E) that comes from trans fatty acids, there is a 23% increase (95% confidence interval 11% to 37%) in incidence of coronary heart disease.^4^ Higher intake is also associated with increased all cause mortality.^5^ This makes trans fatty acids more dangerous on a per gram basis than other types of fat. 

Elimination of trans fatty acids from the UK diet is a key element of a suite of dietary policies that could reduce mortality from coronary heart disease in the UK.^6^ Elimination is recommended by the UK Faculty of Public Health^7^ and by the World Health Organization, which includes elimination in its global strategy on diet, physical activity and health*.*^8^ Currently in the UK, elimination of trans fatty acids is a component of the Department of Health’s “public health responsibility deal,”^9^ which simply advocates voluntary reformulation. Internationally, however, several types of public health policies have been enacted to reduce consumption of trans fatty acids^10^: total bans, mandatory labelling, restaurant bans, and voluntary reformulation. We evaluated three options for further restricting consumption in England: a ban of trans fatty acids in processed foods, improved labelling of trans fatty acids, and a restaurant ban.

## Methods

For policies aimed at reducing consumption, we calculated health benefits and cost outcomes for 2015-20 in England only. We restricted analysis to England because it is then feasible to easily link a socioeconomic indicator to mortality from coronary heart disease and incidence, where coronary heart disease is defined by ICD (international classifications of diseases) codes I20-I25. We applied methods originally incorporated into the previously validated IMPACT model, which explained between 80% and 95% of the decline in mortality from coronary heart disease from 2000 to 2007 for each socioeconomic circumstance group in England (IMPACT-SEC model) 11 and recently extended into a trans fat model.^12^ The methods have been used to predict improvements in mortality from coronary heart disease that could result from improvements in other dietary and lifestyle risk factors.^6^ ^13^ Briefly, IMPACT-SEC calculates the changes in numbers of patients with coronary heart disease and mortality between two time points attributable to changes in risk factors and treatments. In our analysis, the risk factor was consumption of trans fatty acids. Analyses were stratified by age, sex, and socioeconomic status. Age was categorised in 10 year bands starting at 25-34 and included an open ended final group of ≥85. Socioeconomic status was summarised by fifths of the index of multiple deprivation (IMDQ, where IMDQ1 is most affluent and IMDQ5 is most disadvantaged).^14^ This index aggregates seven domains of deprivation (income, employment, health, education, crime, access to services, living environment), each of which is composed of around five indicators. It is an area based measure, and each small area has around 1500 people. Fifths are formed to have equal numbers of areas; because the populations per area are roughly constant, the populations of the fifths are nearly equal as well. More details of each step in the calculations are outlined the appendix.

### Consumption of trans fatty acids in UK

We determined intake of trans fatty acids from the most recent National Diet and Nutrition Survey,^15^ a representative sample of UK households where selected individuals complete food diaries. Based on the average grams per day of trans fatty acids and the average kcal per day, we used a value of 9 kcal per gram to convert grams of trans fatty acids to a percent energy (%E) basis. The result is that consumption was about 0.7%E in 2008-09 to 2011-12. We treated this as the average consumption for IMDQ3 in England. An early release of data covering 2008-09 to 2009-10 indicated slightly higher consumption (0.8%E). Lower income groups have higher consumption, of about 1.3%E based on data released in 2007.^16^ We treated this as the consumption for the most disadvantaged fifth for IMDQ5 in England, with a slight decrease to 1.2%E to account for differences in the time periods. 

The National Diet and Nutrition Survey estimates which food types contribute trans fatty acids, with the primary sources of industrial trans fatty acids being cereal products, margarines, potato chips, confectionary, and other snacks. We estimated a level of trans fatty acids from ruminant sources to be at most 0.4%E. The way in which data are reported (such as “pork and dishes”) implies that some of this might still be industrial trans fatty acids (such as the breading on meat in microwavable meals).

Because there are no data for consumption of trans fatty acids for more affluent socioeconomic groups, we assumed that IMDQ1 had a value of 0.5%E, implying that nearly all of their consumption is from ruminant sources. The total consumption and ruminant consumption for all IMDQs are summarised in table 3, and details from the dietary surveys are in table A and B in the appendix, in which we also show the key results if consumption is equal across IMDQs.

### Policy options modelled

We modelled three policy options that could reduce consumption of trans fatty acids in England, based on an international policy review.^10^ Our comparator was that future consumption would remain constant, therefore not effecting underlying trends in mortality from coronary heart disease.

#### Total ban in processed foods

Beginning in 2004, Denmark required that oils and fats used in processed foods contain less than 2% trans fatty acids. Because this was at the level of ingredients, rather than in the final product, consumption of industrial trans fatty acids was reduced to essentially zero.^17^ This reduction made an important contribution to declines in coronary heart disease in Denmark.^18^ Bans on trans fatty acids in Austria and Switzerland began in 2009,^19^ Iceland in 2010, and Hungary and Norway in 2014.^20^ The United States has initiated the process to ban trans fatty acids in processed foods.^21^

We evaluated the effect of a total ban in processed foods in England by lowering consumption to the baseline for ruminant trans fatty acids (0.4%E as outlined above). For example, this would mean reducing intake from 0.7%E to 0.4%E, a reduction of 0.3%E.

#### Labelling

In the US, the Food and Drug Administration requires that trans fatty acids be listed on food labels. Products with less than 0.5 g per serving can be labelled as having “zero” trans fatty acids, which is potentially problematic for customers who eat many items with less than 0.5 g per serving.^22^ The rule was announced in 2003 and took effect in 2008.

In a market analysis in the US, trans fatty acid content was reduced in 66% of processed foods between 2007 and 2011. The mean content declined by 49% between 2007 and 2011 because of voluntary reformulation on the part of industry. The decline was greatest in the first year (30% in 2007-08.)^23^

We evaluated two labelling scenarios for England. We modelled a 49% reduction in trans fatty acids in processed foods based on the experience in the US.^23^ To evaluate potential socioeconomic gradients, we assumed that IMDQ1 would have the full benefit and IMDQ5 would have only half that reduction (24.5%) based on expert opinion of socioeconomically differentiated response to salt labelling.^24^ Other IMDQs were linearly interpolated between IMDQ1 and IMDQ5. To estimate the maximum effect that labelling might have, we assumed that the maximum response of 49% reduction in trans fatty acids in processed foods would occur in all IMDQs.

#### Partial ban: restaurant and takeaway (fast food) ban

In July 2008, New York City implemented a policy that required restaurant items to contain a maximum of 0.5 g trans fatty acids per serving. This was initially a voluntary ban but, because of poor compliance, became mandatory after 12 months. In a before and after analysis of major chain fast food restaurants, there was an 83% decline (more than 2 g) per serving. The corresponding increase in saturated fat per serving was much smaller (0.5 g).^25^ As with labelling, quantities less than 0.5 g per serving could still lead to non-trivial accumulations, especially among heavy consumers.

We evaluated two restaurant scenarios for England. There are no data from the UK about consumption of trans fatty acids in restaurant food. As a proxy, we used data on food expenditure away from home (excluding alcohol),^26^ assuming that consumption away from home is proportional to expenditure away from home. Data are given by 10th of household income. We converted this to IMDQ (for example, the two lowest 10ths of income were assigned to IMDQ5). With these assumptions, people in IMDQ1 consume 40% of trans fatty acids outside the home, with an approximately linear decline to 22% for IMDQ5. This scenario reflects restaurant food. Details are in table C in the appendix. We ran a second scenario for takeaway/fast food. Expenditure data might be a poor proxy for consumption of trans fatty acids because fast food consumption is likely to be higher in the most disadvantaged groups. This scenario tested the reverse, whereby people in IMDQ5 consume 40% of trans fatty acids away from home, declining to 22% for IMDQ1.

### Health outcomes

We calculated the reduction in incidence of coronary heart disease based on the meta-analysis^4^ relating consumption of trans fatty acids to incidence of coronary heart disease (23% (95% confidence interval 11% to 37%) reduction per 2%E decrease in trans fatty acids). We assumed that mortality from coronary heart disease would change by the same amount and that reducing trans fatty acids would have no effect on case fatality. To create age specific values for the benefit between trans fatty acids and mortality from coronary heart disease, we assumed that the association would resemble that observed for cholesterol and coronary heart disease.^27^ Details can be found in table D in the appendix.

A projection of mortality from coronary heart disease for England for 2015-20 was estimated using an age-period-cohort model.^3^ This model extrapolated past trends in age specific mortality from coronary heart disease by sex and IMDQ. 

We used reductions in mortality from coronary heart disease to calculate deaths prevented or postponed for each year 2015-20. For example, if mortality was reduced from 50 per 100 000 to 45 per 100 000, then there would be five deaths prevented or postponed per 100 000. These rates were converted to absolute numbers by using population projections from UK’s Office for National Statistics (ONS). We further stratified these into IMDQ using the age-sex structure from 2013 (described in the appendix).

Deaths prevented or postponed were translated to life years gained by partitioning them in 2020 into three disease states: diagnosed coronary heart disease, undiagnosed coronary heart disease, and no coronary heart disease. The percentages attributed to each disease state were 26%, 26%, and 48%, respectively, based on the contribution of treatments to declines in mortality from coronary heart disease for the IMPACT-SEC model.^11^ Each disease state has its own median survival stratified by age and sex (table E in the appendix). The deaths prevented or postponed for each disease state were multiplied by the median survival for each disease state and then summed to yield the total life years gained. Smolina and colleagues describe the method in more detail.^28^ A social gradient in median survival by IMDQ for undiagnosed coronary heart disease and no coronary heart disease was applied based on recent analyses from the Office for National Statistics.^29^ We assumed no differences in median survival for diagnosed coronary heart disease because treatment uptake has been shown to be equitable.^30^

To further convert life years gained to quality adjusted life years (QALYs), we used health state utility values^31^ for each of the three subgroups of coronary heart disease (acute myocardial infarction, unstable angina, and heart failure). The proportions of incident cases of total coronary heart disease in each subgroup were based on data from British Heart Foundation.^1^ For each subgroup, we multiplied the health state utility value by the life years gained, weighted by the incidence proportion. Table 1 shows these values.

**Table 1 tbl1:** Health state utility values* and direct health costs per patient.^38^ Proportions of patients in each subtype of coronary heart disease (CHD) are based on data from British Heart Foundation^1^

CHD subtype	Health state utility values	Cost per patient	Proportion
Acute myocardial infarction	0.88	£2880	0.69
Unstable angina	0.80	£2638	0.14
Heart failure	0.71	£2974	0.17

*1 represents perfect health, 0 represents death, and intermediate values signify that life years lived after CHD incidence are hampered by poor health.^32 ^QALY per life year gained is average of health state utility values weighted by proportions, which works out to 0.84.

### Inequality in health outcomes

We used the slope index^32^ to quantify absolute inequality in deaths prevented across IMDQ. Based on a simple linear regression of the number of deaths by IMDQ, the slope index is the fitted difference between IMDQ5 and IMDQ1. This takes into account variation across all IMDQs, which can be slightly different than the simple difference between IMDQ5 and IMDQ1. We calculated the slope index at baseline (without trans fatty acids policies) and with each policy option, and we report the change in the index. For example, if the baseline slope index of deaths prevented or postponed is 10 000 and the slope index with a trans fatty acids policy is 8000, then the change in slope index is −2000 (an absolute inequality reduction of 2000 deaths from coronary heart disease). An illustrative example is shown in the appendix.

### Economic modelling

The cost utility analysis considered the societal costs, including costs to the state and industry, and savings in costs for healthcare and wider economy. The state costs included a one-off cost associated with passing legislation and an ongoing cost of policy monitoring. The industry costs included a one-off cost to industry of product re-formulation as well as an ongoing “lost profitability” cost. The wider economy benefits included the saving from averted productivity loss and saved informal care costs. All costs and benefits were modelled over the five year period 2015-20 for each of the policy scenarios outlined above.

#### State costs

Government costs because of legislation (a one-time cost of £5m in 2015) were estimated from the salt policy analysis^33^ to include such activities as consultancy fees and implementation. Monitoring costs (annual cost of £2.4m) have been reported by the Food Standards Agency^34^ to capture activities related to checks on labelling and updates to collection of data from dietary surveys. 

#### Industry costs

Worst case industry costs for reformulation are assumed to be £200m (8000 products at £25 000 per product).^34^ ^35^ The best case would be zero if reformulation is already built into the business model and occurs about every 18-36 months.^36^ For policies that lead to partial reformulation, the worst case scenario was assumed to be proportional to the average reduction in trans fatty acids (for example, an average 50% reduction would have worst case costs of 50% of £200m—that is, £100m). For labelling, we assumed a baseline of £20m from changes in packaging and loss through disuse of existing packaging,^33^ with reformulation then added on top. All policies would also have ongoing (annual) costs to industry estimated at about £2m.

### Healthcare costs and savings

We created a counterfactual for the number of patients with coronary heart disease in three major categories (acute myocardial infarction, unstable angina, heart failure) using a baseline of numbers of patients in 2007.^11^ Baselines for future years were estimated by assuming that numbers (incidence) would continue to decline by 5% annually, as observed between 2002 and 2010.^1^ Then, based on the reduction in trans fatty acids for each policy option, we calculated that incidence would decline using the trans fatty acids meta-analysis.^4^ Direct healthcare costs per patient group were reported by the Personal Social Services Research Unit^37^ (table 1).

#### Informal care savings

Informal care savings per patient were calculated from the total informal care expenses in 2009 divided by the total number of patients with coronary heart disease in 2009^1^ (£1.4bn/126 000 patients=£12 200 per patient in 2009). Informal care represents the opportunity cost that carers incur by not participating in paid work, based on those caring for patients with coronary heart disease who were the most severely hampered by the disease.^38^

#### Productivity loss averted

Productivity loss averted was calculated with the frictional unemployment savings method, representing the time for an ill employee to be replaced by a new employee as opposed to the time away from work lost by the ill employee. Frictional unemployment is the product of the average time to replace an employee, the average hours worked a day, and the average hourly wage.^39^ For 90 days at eight hours a day this works out at about £9100 per patient with coronary heart disease. Frictional unemployment is applied only to the working age population (age <65).

#### Cost effectiveness

We calculated net costs of each modelled policy at three levels. Firstly, we summed all cost savings (healthcare plus informal care plus averted productivity loss; see table 6). Next, we added on the fixed costs common to all policies (costs to government plus ongoing expenses to industry; see table 7), representing the optimistic estimate of the net costs of each policy. Finally, we added on the worst case costs to industry, representing the pessimistic estimate of net costs of each policy (see table 7).

#### Inflation and discounting

Economic parameters from past data (for example, cost per patient from 2011) were inflated to 2015 by assuming 3% inflation annually, which is about the average annual increase in the consumer price index since 2008.^40^ Costs and benefits for beyond 2015 were discounted back to 2015 at a rate of 3.5% annually according to NICE guidelines.^31^ QALY were discounted back to 2015 with this same rate.

### Sensitivity analysis

We used probabilistic sensitivity analysis to estimate the effect of uncertainty in key parameters. For each policy option, we performed 1000 runs of the full model in R version 3.0.1.^41^ The key parameters that we included were consumption of trans fatty acids, link to mortality from coronary heart disease, mortality from coronary heart disease, numbers of patients with coronary heart disease (incidence), and, for labelling, the strength of the gradient of socioeconomic class (table 2). We also present in the appendix a one way sensitivity analysis of the gradient socioeconomic class in consumption of trans fatty acids.

**Table 2 tbl2:** Model parameters included in probabilistic sensitivity analysis of effect of changes in consumption of trans fatty acids (TFA) on coronary heart disease (CHD)

Model parameter	Sensitivity analysis
TFA consumption	Normal distribution with SE of mean of 0.02%E estimated from National Diet and Nutrition Survey.^15^ If this caused values to fall below minimum for ruminant TFA (that is, <0.4%E), values were set at 0.4%E
TFA link to incidence of CHD	Normal distribution of meta-analysis coefficient (23%, 95% CI 11% to 37%)
Labelling response gradient	PERT distribution* with mean 50%, min 20% and max 105%^24^
CHD mortality	Normal distribution of logit of predicted rates based on upper and lower confidence intervals from model output^3^
No with CHD (incidence)	Annual percent decline in patient numbers could vary from 0% to 10%. PERT distribution was used with best estimate of 5% decline
Direct health costs per patient	Assume PERT distribution with 20% variability around reported values (table 1)
Median survival	Assume PERT distribution with 20% variability around medians

*PERT distribution is form of β distribution based on best, minimum, and maximum estimates for parameter.^42^

### Patient involvement

There was no patient involvement in this study.

## Results

### Reduction in consumption of industrial trans fatty acids 

A full ban on industrial trans fatty acids in processed foods could halve the amount of trans fatty acids in the English diet, resulting in a new average of around 0.4% of energy from ruminant trans fatty acids (meat, dairy, etc). Improved labelling and associated voluntary reformulation could be at best half as effective as a total ban. If labelling also has a behavioural component that favours the more affluent (IMDQ1), then the most disadvantaged would see little decline in consumption of trans fatty acids (a decline of 0.20% energy for IMDQ5). Bans on trans fatty acids in restaurants would favour the most affluent based on our proxy of food expenditure. The effect would be about 40% of that of the full ban for the most affluent and about 20% for the most disadvantaged. Alternatively, if trans fatty acids are mostly consumed in fast food outlets used by the most disadvantaged, they would proportionally benefit more but still fall well short of the potential of a full ban. Table 3 provides detailed results for all policies[Table tbl3].

**Table 3 tbl3:** Baseline consumption of trans fatty acids (%E) and reductions associated with modelled policy options stratified by fifths of index of multiple deprivation (IMDQ). Maximum reduction is down to 0.4%E based on ruminant trans fatty acids remaining in diet. Numbers in parenthesis are 95% confidence intervals from probabilistic sensitivity analysis

IMDQ	Total consumption*	Ruminant sources†	Reductions				
			Total bans‡	Label (no SEC gradient)§	Fast food¶	Label (SEC gradient)**	Restaurant††
1	0.50 (0.46 to 0.54)	0.40	0.10 (0.06 to 0.14)	0.05 (0.03 to 0.07)	0.02 (0.01 to 0.03)	0.05 (0.04 to 0.06)	0.04 (0.02 to 0.06)
2	0.60 (0.56 to 0.64)	0.40	0.20 (0.16 to 0.24)	0.10 (0.08 to 0.12)	0.05 (0.04 to 0.06)	0.09 (0.08 to 0.10)	0.07 (0.05 to 0.09)
3	0.70 (0.66 to 0.74)	0.40	0.30 (0.26 to 0.34)	0.15 (0.13 to 0.17)	0.10 (0.09 to 0.11)	0.11 (0.10 to 0.12)	0.10 (0.09 to 0.11)
4	0.95 (0.91 to 0.99)	0.40	0.55 (0.51 to 0.59)	0.27 (0.25 to 0.29)	0.19 (0.17 to 0.21)	0.17 (0.15 to 0.19)	0.15 (0.14 to 0.16)
5	1.20 (1.16 to 1.24)	0.40	0.80 (0.76 to 0.84)	0.39 (0.37 to 0.41)	0.32 (0.30 to 0.34)	0.20 (0.18 to 0.22)	0.18 (0.17 to 0.19)
Average	0.79 (0.75 to 0.83)	0.40	0.39 (0.35 to 0.43)	0.19 (0.17 to 0.21)	0.14 (0.12 to 0.16)	0.12 (0.10 to 0.14)	0.11 (0.10 to 0.12)

*Estimated from dietary surveys.^15 16^

†Consumption from ruminant sources.^15 16^

‡Eliminate all trans fatty acids from processed foods—that is, reduce to 0.4%.

§Reduce by 49% of total ban amount for all IMDQ.^23^

¶Reverse IMDQ gradient of expenditure away from home (favours IMDQ5)††.

**IMDQ1 has maximum label effect (49%), linear down to IMDQ5 at 50%^24^ of that (50% of 49% = 24.5%).

††Consumption away from home proportional to food spending away from home (favours IMDQ1).

### Health benefits of reduction in trans fatty acids

We translated the reduction in trans fatty acids to a reduction in deaths from coronary heart disease over the period 2015-20 (table 4). A full ban would reduce total coronary heart disease deaths by about 2.6% (7200 deaths, 95% confidence interval 3200 to 12 500 deaths), and the disadvantaged would benefit more because of their higher mortality from coronary heart disease and greater reduction in intake of trans fatty acids. Inequality in deaths from coronary heart disease would be reduced by about 15% (3000 deaths, 1300 to 5200 deaths). Labelling with no social gradient would be about half as effective across all groups. If response to labelling has a social gradient, as seems plausible, the reduction would be around a third compared with the total ban. In the fast food scenario, where we assumed that consumption of trans fatty acids outside the home is higher in the poorest fifth, the effect would be slightly less than that for labelling and around a third as effective as the total ban. A restaurant ban that favours the more affluent would be about a quarter as effective as the total ban.

**Table 4 tbl4:** Total deaths from coronary heart disease for 2015-20 and reductions associated with each policy option, stratified by index of multiple deprivation (IMDQ). Figures are baseline values and reductions* (95% confidence intervals from probabilistic sensitivity analysis); % reductions. Absolute inequality in coronary heart disease mortality across IMDQs is measured by slope index^33^

IMDQ	Baseline	Reductions				
		Total ban	Label (no SEC gradient)	Fast food	Label (SEC gradient)	Restaurant
1	43 000	300 (100 to 400); 0.7%	100 (100 to 200); 0.2%	100 (0 to 100); 0.2%	100 (100 to 200); 0.2%	100 (0 to 200); 0.2%
2	50 000	600 (300 to 1100); 1.2%	300 (100 to 500); 0.6%	200 (100 to 300); 0.4%	300 (100 to 400); 0.6%	200 (100 to 400); 0.4%
3	57 000	1000 (500 to 1800); 1.8%	500 (200 to 900); 0.9%	300 (200 to 600); 0.5%	400 (200 to 700); 0.7%	300 (100 to 600); 0.5%
4	59 000	2000 (900 to 3600); 3.4%	1000 (400 to 1800); 1.7%	700 (300 to 1300); 1.2%	600 (300 to 1100); 1.0%	500 (200 to 900); 0.8%
5	64 000	3300 (1400 to 5600); 5.2%	1600 (700 to 2800); 2.5%	1300 (600 to 2300); 2.0%	800 (300 to 1400); 1.3%	700 (300 to 1300); 1.1%
Total	273 000	7200 (3200 to 12 500); 2.6%	3500 (1500 to 6200); 1.3%	2600 (1200 to 4600); 1.0%	2200 (1000 to 3800); 0.8%	1800 (700 to 3400); 0.7%
Absolute inequality	20 400	3000 (1300 to 5200); 14.7%	1500 (600 to 2600); 7.4%	1200 (600 to 2200); 5.9%	700 (200 to 1200); 3.4%	600 (300 to 1100); 2.9%

*For example, reduction of 3000 means slope index of total ban would be 17 400.

We translated the reduction in mortality in the year 2020 into QALYs (table 5). Again, the total ban would be especially beneficial for the most disadvantaged (IMDQ5) because the differences in mortality from coronary heart disease across IMDQs are greater at younger ages, when deaths avoided save more life years. As before, the other policy scenarios would be about a third to a half as effective as the total ban.

**Table 5 tbl5:** Gain in quality of life years (QALY) in 2020 associated with each policy option (95% confidence intervals from probabilistic sensitivity analysis), stratified by index of multiple deprivation (IMDQ)

IMDQ	Total ban	Label (no SEC gradient)	Fast food	Label (SEC gradient)	Restaurant
1	200 (100 to 400)	100 (0 to 200)	100 (0 to 100)	100 (0 to 200)	100 (0 to 200)
2	600 (200 to 1000)	300 (100 to 500)	200 (100 to 300)	200 (100 to 400)	200 (100 to 400)
3	1000 (400 to 1800)	500 (200 to 900)	300 (100 to 600)	400 (100 to 700)	300 (100 to 600)
4	2200 (800 to 3900)	1100 (400 to 2000)	800 (300 to 1400)	700 (200 to 1200)	600 (200 to 1100)
5	3900 (1500 to 6800)	2000 (700 to 3500)	1600 (600 to 2800)	1000 (300 to 1700)	900 (300 to 1600)
Total	7900 (3000 to 13 900)	4000 (1400 to 7100)	3000 (1100 to 5200)	2400 (700 to 4200)	2100 (700 to 3900)

### Economic benefits and costs 

Table 6 shows the potential direct healthcare savings, averted productivity loss, and informal care savings for 2015-20. The total ban would save about £42m in direct health expenses, with nearly half of that (£19m) attributable to the most disadvantaged fifth (IMDQ5). As with QALYs, the most disadvantaged would stand to gain proportionally more in averted productivity loss because the incidence of coronary heart disease is higher among the most disadvantaged at younger ages compared with the most affluent. The informal care savings are the largest component of these savings, representing about £196m savings from 2015 to 2020.

**Table 6 tbl6:** Direct healthcare savings (£m), averted productivity loss, and informal care savings for 2015-20 with each policy option, stratified by index of multiple deprivation (IMDQ). Direct healthcare savings include patients with acute myocardial infarction, unstable angina, and heart failure. Averted productivity loss due to lower incidence of coronary heart disease (among those aged <65) is based on frictional unemployment period. Informal care savings are based on per patient informal care costs^1 39^ (95% confidence intervals from probabilistic sensitivity analysis)

IMDQ	Total ban	Label (no SEC gradient)	Fast food	Label (SEC gradient)	Restaurant
**Healthcare savings(2015-2020)**					
1	1.6 (0.7 to 2.5)	0.8 (0.3 to 1.2)	0.4 (0.2 to 0.6)	0.8 (0.3 to 1.2)	0.6 (0.3 to 1.0)
2	3.7 (1.6 to 5.7)	1.8 (0.8 to 2.8)	1.0 (0.4 to 1.5)	1.6 (0.7 to 2.5)	1.3 (0.6 to 2.0)
3	5.9 (2.6 to 9.3)	2.9 (1.3 to 4.6)	1.9 (0.8 to 3.0)	2.2 (1.0 to 3.4)	1.9 (0.8 to 3.0)
4	11.6 (5.1 to 18.2)	5.8 (2.5 to 9.0)	4.1 (1.8 to 6.4)	3.6 (1.6 to 5.7)	3.1 (1.4 to 4.9)
5	19.2 (8.4 to 30.1)	9.6 (4.2 to 15.0)	7.9 (3.5 to 12.3)	4.8 (2.1 to 7.6)	4.4 (1.9 to 6.9)
Total	41.9 (18.5 to 65.8)	20.8 (9.2 to 32.7)	15.2 (6.7 to 23.8)	13.0 (5.7 to 20.4)	11.3 (5.0 to 17.8)
**Averted productivity loss (2015-20)**					
1	1.8 (0.8 to 2.8)	0.9 (0.4 to 1.4)	0.4 (0.2 to 0.6)	0.9 (0.4 to 1.4)	0.7 (0.3 to 1.1)
2	4.3 (1.9 to 6.7)	2.1 (0.9 to 3.3)	1.1 (0.5 to 1.8)	1.9 (0.8 to 2.9)	1.5 (0.7 to 2.3)
3	7.4 (3.3 to 11.6)	3.7 (1.6 to 5.7)	2.4 (1.1 to 3.8)	2.7 (1.2 to 4.3)	2.4 (1.1 to 3.8)
4	15.8 (7.0 to 24.9)	7.9 (3.5 to 12.4)	5.6 (2.4 to 8.7)	5.0 (2.2 to 7.8)	4.3 (1.9 to 6.7)
5	29.8 (13.1 to 46.7)	14.9 (6.6 to 23.4)	12.3 (5.4 to 19.3)	7.5 (3.3 to 11.8)	6.9 (3.0 to 10.8)
Total	59.1 (26.0 to 92.7)	29.4 (13.0 to 46.2)	21.8 (9.6 to 34.2)	18.0 (7.9 to 28.2)	15.8 (6.9 to 24.8)
**Informal care savings (2015-20)**					
1	7.3 (3.2 to 11.4)	3.6 (1.6 to 5.6)	1.6 (0.7 to 2.6)	3.6 (1.6 to 5.6)	2.9 (1.3 to 4.6)
2	17.0 (7.5 to 26.7)	8.4 (3.7 to 13.1)	4.5 (2.0 to 7.1)	7.3 (3.2 to 11.5)	5.9 (2.6 to 9.2)
3	27.5 (12.1 to 43.3)	13.6 (6.0 to 21.3)	8.9 (3.9 to 14.0)	10.2 (4.5 to 16.0)	8.9 (3.9 to 14.0)
4	54.2 (23.9 to 85.1)	26.9 (11.8 to 42.2)	18.9 (8.3 to 29.7)	16.9 (7.4 to 26.5)	14.5 (6.4 to 22.8)
5	89.7 (39.5 to 140.8)	44.7 (19.7 to 70.2)	36.8 (16.2 to 57.7)	22.6 (9.9 to 35.4)	20.6 (9.1 to 32.3)
Total	195.7 (86.1 to 307.3)	97.1 (42.7 to 152.5)	70.8 (31.1 to 111.1)	60.5 (26.6 to 95.0)	52.9 (23.3 to 83.0)
Grand total	296.7 (130.6 to 465.9)	147.4 (64.9 to 231.4)	107.7 (47.4 to 169.1)	91.5 (40.2 to 143.6)	80.0 (35.2 to 125.6)

The costs to government would amount to about £5m for legislation and some £16.6m for annual monitoring, a sum of about £21.6m (table 7). Ongoing expenses to industry would amount to about £11m. The sum of these costs, which are common to all policy options, is about £32.6m. If industry reformulation costs are zero, this £32.6m would be the cost for each policy, which is our best case scenario. If industry incurred substantial reformulation costs, the worst case costs might lie between £48m and £200m, according to the proportion of the total product line that required reformulation (table 3 shows percent reductions in consumption of trans fatty acids).

**Table 7 tbl7:** Policy costs (£m) to government for legislation (one time) and monitoring (annually) of reduction in trans fatty acids . Costs to industry associated with possible lost profits (annually) and reformulation (one time). If reformulation occurs as part of normal product life cycle, there would be no incremental cost for reformulation, reflected in optimistic net cost. Pessimistic net cost includes reformulation costs to industry. Negative net costs represent savings

	Common to all policies	Industry costs (worst case)				
		Total ban	Label (no SEC gradient)	Fast food	Label (SEC gradient)	Restaurant
Government:						
Legislation	5.0	—	—	—	—	—
Monitoring	16.6	—	—	—	—	—
Industry:						
Ongoing expenses	11.0	—	—	—	—	—
Reformulation		200.0	92.9	62.6	56.1	47.4
Sum all costs	32.6	232.6	125.5	95.2	88.7	80.0
Sum all savings	—	296.7 (130.6 to 465.9)	147.4 (64.9 to 231.4)	107.7 (47.4 to 169.1)	91.5 (40.2 to 143.6)	80.0 (35.2 to 125.6)
Optimistic net cost	—	−264.1 (−433.3 to −98.0)	−114.8 (−198.8 to −32.2)	−75.1 (−136.5 to −14.8)	−58.9 (−111.0 to −7.6)	−47.4 (−93.0 to −2.6)
Pessimistic net cost	—	−64.1 (−233.3 to 102.0)	−21.9 (−105.9 to 60.6)	−12.5 (−73.9 to 47.8)	−2.8 (-54.9 to 48.4)	0.0 (−45.5 to 44.8)

### Cost effectiveness of policy options

The sum of all savings for the total ban would be about £297m (95% confidence interval £131m to £466m), while for other policies the best estimates would lie in the range of £80-147m (fig 1). In the optimistic scenario, where the only costs would be to government and for ongoing expenses to industry, the costs might amount to £33m (table 7). The net costs would be a savings of £264m (£98m to £433m) for the total ban or in the range £48m to £115m for other policies (table 7).

**Figure fig1:**
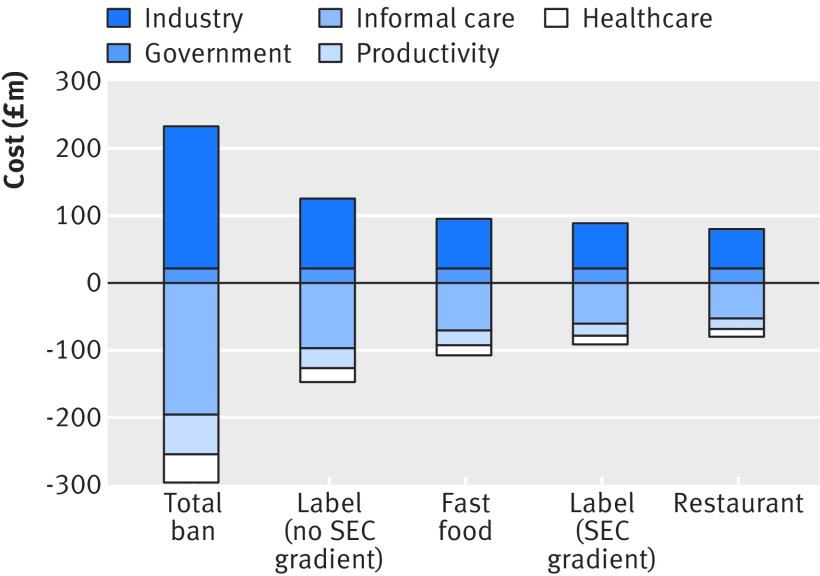
**Fig 1** Contributions of each cost category for each policy option for reduction in trans fatty acids. Costs to government and industry are expressed as positive and savings from reductions in direct healthcare, productivity loss, and informal care are expressed as negative. (Confidence intervals on savings are shown in table 6; net costs are shown in table 7 and fig 2)

In the pessimistic scenario, where industry incurs substantial reformulation costs outside the normal business cycle, the costs might amount to a total of £233m for the total ban and between £80m and £126m for the other policy options. The net costs for the total ban would be savings of about £64m (95% confidence interval −£102m to £233m), while for other policies the net costs would save in the range of £0-£22m. 

Figure 2 summarises the net costs and the reduction in absolute inequality of coronary heart disease mortality for each policy option. The additional benefits to the most disadvantaged group are evident in the large reduction in absolute inequality (reduction of 3000 coronary heart disease deaths). Combined with table 4, we can conclude that the larger reductions in incidence of coronary heart disease for IMDQ4 and IMDQ5 are the primary cause of cost saving.

**Figure fig2:**
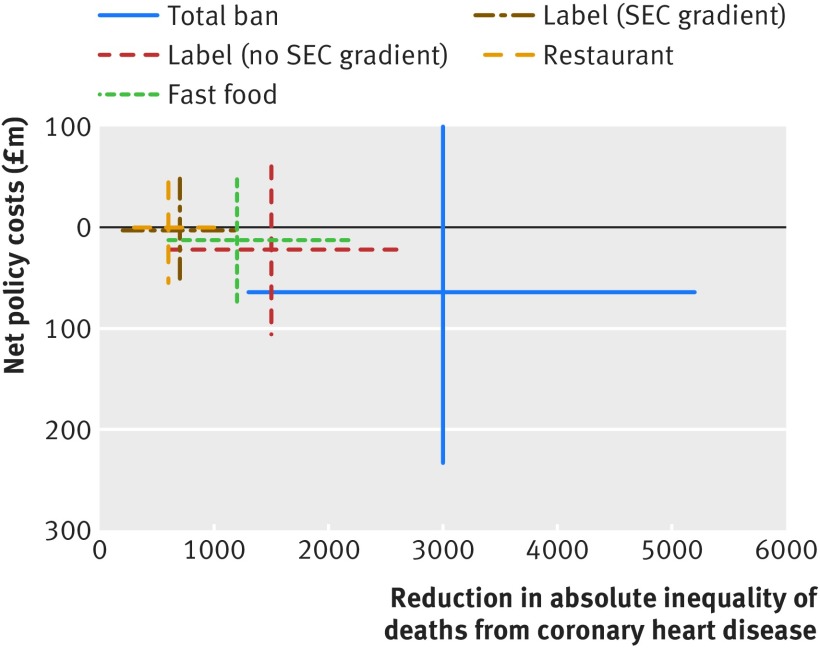
**Fig 2** Net costs (negative is saving) versus reduction in inequality of coronary heart disease mortality (positive is reduction). Net costs are from pessimistic scenario (table 7), where industry reformulation costs are substantial. Reduction in absolute inequalities is change in slope index with and without each policy option. Total ban separates from other policy options along both cost effectiveness and inequality reduction axes. In fig B in the appendix, the optimistic scenario is presented, which excludes reformulation costs to industry

## Discussion

Policies to reduce or eliminate industrial trans fatty acids could powerfully improve coronary heart disease mortality, incidence, and inequalities in England. A total ban might reduce mortality from coronary heart disease for 2015-20 by around 2.6%, representing 7200 fewer deaths (95% confidence interval 3200 to 12 500). Absolute inequality in mortality from coronary heart disease might be reduced more substantially, by nearly 15%, representing almost 3000 fewer deaths (1300 to 5200). The other modelled policies might reduce the number of deaths by 1800 to 3500, thus being at most half as effective as the total ban. The reduction in absolute inequality of deaths from coronary heart disease would similarly be at most half as much, with a reduction in the range of 600 to 1500. Labelling with no socioeconomic gradient would be the most effective policy after the total ban, followed by the fast food ban. Reduction in trans fatty acids is thus key to reducing inequality in mortality from coronary heart disease because more disadvantaged individuals have higher consumption^16^ and higher mortality from coronary heart disease.^2^

If reformulation costs to industry occur as part of the regular business cycle, any policy we modelled would be dominant over a “do nothing” scenario, where consumption of trans fatty acids remains constant, even when we consider uncertainty in the estimates. If reformulation costs to industry are substantial, these policy options would still be dominant over a “do nothing” scenario based on the best estimates, though the uncertainty leaves some possibility of net losses in the short term horizon that we focused on.

The policies we modelled vary in their assumed coverage^43^: 100% coverage for a total ban, at most 49% for labelling, and at best 40% for a restaurant/fast food ban. There would likely be socioeconomic gradients in response to labelling and place specific bans, further reducing the coverage among the most disadvantaged groups. By failing to reach those individuals who stand to benefit most, these policies might be only a third to a half as effective.

Public health policies vary in whether they change the environment (structural policies) or rely on individual behaviour change (agentic policies).^44^ Agentic policies generally widen health inequalities, whereas structural policies can reduce inequalities or be neutral.^45^ ^46^ The policies we modelled are all structural in the sense that they would change the food environment to restrict consumption of trans fatty acids. The reduction in coverage among the most disadvantaged stems from an agentic element to labelling and restaurant bans. We did not model any purely agentic policies because there is little evidence of their effectiveness in dissuading people from consuming products with trans fatty acids. Our results are also consistent with the idea that structural interventions need to be inter-sector in nature, not just relying on healthcare to reach vulnerable populations.^47^

Recent downward trends in consumption of trans fatty acids in the UK^48^ are a result of voluntary reformulation of some products (such as biscuits/cookies).^49^ Complete elimination in countries such as Denmark, however, shows that continued reformulation is technically feasible.^9^ ^19^ In fact Denmark showed the largest decline in mortality from coronary heart disease in the EU from 1980 to 2009, but the precise contribution of the ban to this trend has not been quantified.^50^ Furthermore, lowering average consumption with less stringent policies might still conceal small groups of consumers with extremely high consumption. In Denmark before the ban, an estimated 1% of the population were consuming around 5 g/day of trans fatty acids, with potentially large mortality consequences.^51^

### Strengths and limitations

We used data from a meta-analysis directly linking consumption of trans fatty acids to incidence of coronary heart disease. This removes uncertainty that would accompany modelling a link mediated by cholesterol, and it therefore also includes non-cholesterol effects associated with inflammation. The effects should be even greater if trans fatty acids are replaced with *cis* unsaturated fats.^4^ Generally, foods can be reformulated to remove trans fatty acids without substantially increasing saturated fats,^9^ ^25^ though this might vary by product type.^49^ ^52^

There is debate about whether ruminant trans fatty acids are harmful. While there are no proved links between consumption of ruminant trans fatty acids and mortality from coronary heart disease,^53^ ruminant trans fatty acids increases cholesterol concentration, as does industrial trans fatty acids.^54^ Additional health gains and cost savings might be possible by shifting to a plant based diet, but we did not model this because we focus on trans fatty acids in processed foods.

Our health outcomes analysis assumes continuing declines in incidence of and mortality from coronary heart disease. If future mortality in England plateaus or increases, elimination of trans fatty acids could have even greater benefits. In the case of mortality, the declines are likely to be rapid enough to counteract population ageing.^55^ We assumed that mortality would decline in the same proportion as incidence, rather than additionally modelling changes in case fatality rates. Declines in mortality from coronary heart disease have been shown to depend more on incidence than on case fatality.^56^

We used an area based measure of socioeconomic status (IMDQ). Within an area there will naturally be mixing of individuals with higher and lower status. Therefore, we cannot make firm conclusions about individuals. The advantage of IMDQ is that it allows us to model coronary heart disease mortality for the whole of England without the need to infer from a sample. Further, the dietary surveys we used^15^ ^16^ are the most detailed source of consumption of trans fatty acids for our population. Other sources report higher total food energy intake^57^ but do not indicate the consumption of trans fatty acids. As the effect of trans fatty acids operates on a percentage basis (food energy from trans fatty acids divided by total food energy), differences between surveys would affect the results only if consumption were substantially misreported in the surveys we used.

### Conclusions

Elimination of trans fatty acids from processed foods is an achievable target for public health policy. Dietary changes can have a rapid effect on coronary heart disease.^58^ ^59^ A total ban on trans fatty acids in England is technically feasible and one that neighbouring countries have already accomplished.^9^ ^19^ ^60^ Such a ban would lead to health benefits at least twice as large as other policy options, both in terms of total population benefit and reduction in inequality. Continuing to rely on industry cooperation via the responsibility deal might be insufficient. Furthermore, if attention shifts away from trans fatty acids, these toxic substances could creep back into processed foods. The current policy climate is ideal for shutting the door effectively on trans fatty acids. Decisive action is now indicated, prioritising the most effective and cost effective policy options.

**What is already known on this topic**

Industrial trans fatty acids (TFA) are commonly added to processed foods to cheaply improve shelf life and palatability. Their removal from processed foods in the UK is part of the Department of Health’s responsibility deal, and other countries have used total bans, labelling, and restaurant bans to achieve bigger reductions in consumption
Trans fatty acids increase risk of mortality from coronary heart disease by increasing LDL cholesterol, decreasing HDL cholesterol, and causing systemic inflammation as well as endothelial dysfunction
Mortality from coronary heart disease and consumption of trans fatty acids are higher among disadvantaged socioeconomic groups

**What this study adds**

A total ban on industrial trans fatty acids in processed foods in England might potentially prevent or postpone about 7200 deaths from coronary heart disease (2.6%) in 2015-20 and reduce inequality in mortality from coronary heart disease by about 3000 deaths (15%)
A total ban would have the greatest net cost savings of £264m if reformulation occurs as a normal part of the business cycle or £64m if substantial reformulation costs are incurred outside the normal cycle
Improved labelling of trans fatty acids or bans in restaurants would be at best half as effective in terms of reducing both mortality and inequalities. These policies could still be cost saving from a societal perspective because of the potentially large reductions in incidence of and mortality from coronary heart disease

## Notes

We thank Marissa Collins and Helen Mason of Yunus Centre for Social Business and Health for providing economic data. We also acknowledge the development of the original IMPACT SEC model. The English SEC project was conducted by M Bajekal and S Scholes. H Hande set up the worksheet template; S Scholes populated the model and was its custodian; M Bajekal ensured the integrity of inputs and outputs and provided SEC related methodological solutions. M O’Flaherty and N Hawkins provided support, clinical expertise, and generated the therapeutic input. The UCL team was led by R Raine, and S Capewell coordinated the overall project.

Contributions: MOF and SC developed the idea. KA and JP designed the analysis. All authors commented on versions of the manuscript for key intellectual content. KA is guarantor.

Ethical approval: Not required.

Transparency: The lead author affirms that the manuscript is an honest, accurate, and transparent account of the study being reported; that no important aspects of the study have been omitted; and that any discrepancies from the study as planned have been explained.

Data sharing: Input data for the model are available from corresponding author on request.

## Notes

Cite this as: *BMJ* 2015;351:h4583
