# Supplementary material for: Potential of trans fats policies to reduce socioeconomic inequalities in mortality from coronary heart disease in England: cost effectiveness modelling study
Source: BMJ. 2015 Sep 15;351:h4583. doi: 10.1136/bmj.h4583 (PMC4569940; doi:10.1136/bmj.h4583)
Supplement: Supplementary file 1 [file allk024146.ww1_default.pdf]

Appendix: Supplementary information [posted as supplied by author]

<u>TFA consumption in the UK</u>

Table A: TFA consumption by Age and Gender [15]. Potential Ruminant TFA categories are: Milk and milk products, Butter, Eggs and egg dishes, Meat and meat products, Fish and fish dishes. Within each of these potential ruminant categories, there still may be non-ruminant TFA. For example, the category Milk and milk products includes ice cream and dairy desserts, which can have TFA. The meat and fish categories include fried or breaded dishes, which can have TFA. The figures below are thus likely a slightly over-estimate of ruminant TFA. Therefore we used 0.4%E as the average ruminant TFA consumption. Since the differences by age and gender are not large, we used rounded values of 0.7%E for the average total TFA consumption for IMDQ3 and 0.4%E for the ruminant TFA consumption of all IMDQs.

	MEN		WOMEN	
	Age 19-64	Age 65+	Age 19-64	Age 65+
Trans Fat (grams per day)	1.5	1.5	1.1	1.2
Trans Fat (kcal per day = grams x 9)	13.5	13.5	9.9	10.8
Total food calories (kcal per day)	1981	1845	1542	1479
Total Trans Fat %E (kcal TF / kcal Total)	0.68%	0.73%	0.64%	0.73%
Percent of Trans Fat potentially from ruminant sources	65%	70%	64%	70%
Ruminant Trans Fat %E (product of two previous rows)	0.44%	0.51%	0.41%	0.51%

Table B: TFA consumption from Low Income Diet and Nutrition Survey [16], which we treated as IMDQ5. As above (Table A), the average across age and gender is similar, therefore we used 1.3% as a simple rounded average. Since this data is slightly older than in Table A, we lowered the average here to 1.2%. It is not possible to calculate the ruminant TFA consumption in exactly the same way due to differences in the categories reported. It seems likely that individuals with low socioeconomic status would be more likely to purchase low cost / low quality products that contain industrial TFA (e.g. cheap ice cream, cheap ready meals) whereas those with higher socioeconomic status would be more likely to purchase the equivalent product without processed TFA (e.g. whole cuts of meat).

	MEN			WOMEN		
	Age 35-49	Age 50-64	Age 65+	Age 35-49	Age 50-64	Age 65+
Trans Fat (grams per day)	3.1	2.7	2.5	2.1	2.1	2.2
Trans Fat (kcal per day = grams x 9)	27.9	24.3	22.5	18.9	18.9	19.8
Total food calories (kcal per day)	1983	1902	1723	1508	1464	1407
Total Trans Fat %E (kcal TF / kcal Total)	1.41%	1.28%	1.31%	1.25%	1.29%	1.41%

Policy options modelled

There are no data on food consumption outside the home in the UK. To approximate the consumption of TFA in restaurant food or fast food, we used food expenditure data (Table C). These UK data are very similar to recent data about the proportion of calories consumed outside the home in the United States, both being around 30%. These data from the USA can be found here: http://www.health.gov/dietaryguidelines/data-table-1.asp. Though we cannot necessarily conclude that the data from the USA are applicable to the UK, it is at least reassuring that they are similar.

Table C: Food expenditure (£ per week) at home and away from home by deciles of household income. Alcohol expenditure is excluded. The percent away from home is based on the sum of food at home and food away from home (e.g. for decile 1, 7.20 / (27.00 + 7.20) = 21%). We assumed that the two lowest income deciles were equivalent to IMDQ5, and so on. For modelling a restaurant ban, we assumed that industrial TFA consumption was proportional to the food expenditure away from home (e.g. 22% of industrial TFA consumed by IMDQ5 was at restaurants). We did a sensitivity analysis by running the reverse gradient (e.g. 40% of industrial TFA consumed by IMDQ5), which we refer to as the "fast food" scenario due to the tendency for unhealthy take-away establishments to be clustered in deprived neighbourhoods (Please find the information at the following from National Obesity Observatory: http://www.noo.org.uk/uploads/doc/vid_15683_FastFoodOutletMap2.pdf)

<table>
<tr><td>Income Decile</td><td>1 (lowest)</td><td>2</td><td>3</td><td>4</td><td>5</td><td>6</td><td>7</td><td>8</td><td>9</td><td>10 (highest)</td></tr>
<tr><td>Food at home</td><td>27.00</td><td>31.30</td><td>37.60</td><td>45.10</td><td>50.20</td><td>50.30</td><td>55.60</td><td>60.50</td><td>67.30</td><td>77.50</td></tr>
<tr><td>Food away from home</td><td>7.20</td><td>9.70</td><td>14.30</td><td>15.20</td><td>19.80</td><td>28.30</td><td>27.70</td><td>33.40</td><td>42.90</td><td>54.70</td></tr>
<tr><td>% away from home</td><td>21%</td><td>24%</td><td>28%</td><td>25%</td><td>28%</td><td>36%</td><td>33%</td><td>36%</td><td>39%</td><td>41%</td></tr>
<tr><td>Equivalent IMDQ</td><td colspan="2">5 (lowest)</td><td colspan="2">4</td><td colspan="2">3</td><td colspan="2">2</td><td colspan="2">1 (highest)</td></tr>
<tr><td>% away from home</td><td colspan="2">22%</td><td colspan="2">26%</td><td colspan="2">32%</td><td colspan="2">34%</td><td colspan="2">40%</td></tr>
</table>

Health Outcomes

We treated the meta-analysis relationship between TFA consumption and CHD incidence [4] as log-linear. A 23% increase in CHD incidence per 2%E from TFA is mathematically: log(1.23) / 2%E = 0.104 per 1%E. Then for a certain reduction in TFA consumption (e.g. a 0.3%E reduction), we calculated the reduction in CHD incidence as: 1-exp(0.104 per 1%E x TFA reduction) = e.g., 1-exp(0.104 x 0.3) = -0.032 (this is a 3.2% reduction).

Age-specific effects are not provided in the meta-analysis. Therefore we assumed that age-specific values would follow the same relationship as those linking cholesterol to CHD reported by the Prospective Studies Collaboration (2007) and adapted for the IMPACT-SEC model by Bajekal et al. (2012) [2], since cholesterol is the primary pathway through which TFA increase CHD risk. We assumed there would be no further stratification in the effect by gender or by IMDQ. If we consider the age group 65-74 as an example, the reduction in CHD incidence per 1%E from TFA would be: log(1.12) / 2E% = 0.057 per 1E%. Again following the above paragraph, a 0.3%E reduction in TFA would result in a reduction of 1.7% in CHD incidence (1 –exp(0.057 * 0.3) = -0.017).

Table D: Calculation of age-specific TFA-Mortality effects. The overall (average) effect is from the meta-analysis by Mozaffarian et al. (2006) [4]. The age gradient of the Cholesterol-Mortality effect was adapted from Prospective Studies Collaboration (2007) for the IMPACT-SEC model [2].

	25-34	35-44	45-54	55-64	65-74	75-84	85+
Overall effect on CHD incidence per 2% Food energy from TFA	+23%	+23%	+23%	+23%	+23%	+23%	+23%
Age-specific Cholesterol effect on CHD Mortality	1.43	1.42	1.00	0.71	0.53	0.50	0.49
Age-specific TFA effect on CHD incidence per 2%E from TFA (product of above two rows)	+33%	+33%	+23%	+16%	+12%	+12%	+11%

We converted the proportional reductions in CHD incidence by first assuming that CHD mortality would reduce by the same amount, e.g. 1.7% reduction in incidence would lead to a 1.7% reduction in mortality. Then to convert the percentage reduction to absolute numbers, we used a projection of future CHD mortality by age group, gender and IMDQ. This projection was done using a Bayesian analysis of an age-period-cohort model which has been previously applied to England & Wales [55] and stratified by IMDQ for England [3].We continue with the example above, now illustrating that Men ages 65-74 in IMDQ5 are predicted in 2020 are predicted to have CHD mortality of 379 per 100,000. Reducing this by 1.7% as above would represent 6 CHD DPP per 100,000 in 2020.

The rates of CHD DPP (per 100,000) were converted to absolute numbers using two pieces of information. First, ONS published population counts by age group, gender, and IMDQ for 2002-2013. We first summed the five-year age bands into ten-year age bands, by gender. For each age-gender group, we calculated the proportion of the population in 2013 in each IMDQ. An example follows for men aged 65-74:

IMDQ	M 65-69	M 70-74	M 65-74 (Sum)	Proportion
5	200,601	146,679	347,280	0.144
4	242,979	173,038	416,017	0.172
3	303,520	214,651	518,171	0.214
2	337,354	235,710	573,064	0.237
1	335,226	228,845	564,071	0.233
		Total Sum	2,418,603	1

We then applied these proportions to ONS constant fertility population projections for 2015-2020. Continuing the example:

Year		2015	2016	2017	...
Projected M 65-74 (ONS)		2,541,495	2,602,704	2,642,573	...
Estimates by IMDQ (Our calculations)	Proportion (see above)				
IMDQ5	0.144	364,926	373,715	379,439	...
IMDQ4	0.172	437,155	447,684	454,541	...
IMDQ3	0.214	544,500	557,614	566,155	...
IMDQ2	0.237	602,182	616,685	626,131	...
IMDQ1	0.233	592,732	607,007	616,306	...

The data sources are:

"Number of deaths and populations broken down by sex, year, the adjusted IMD 2010 quintiles of English Lower Super Output Areas and age group, 2002-2013". Available with reference number 003497 at http://www.ons.gov.uk/ons/about-ons/business-transparency/freedom-of-information/what-can-i-request/published-ad-hoc-data/health/november-2014/index.html [26 November 2014]

"National Population Projections, 2012-based extra variants". Available from: http://www.ons.gov.uk/ons/rel/npp/national-population-projections/2012-based-extra-variants/index.html ; Specifically, the following ZIP file [caution: might download automatically] : http://www.ons.gov.uk/ons/rel/npp/national-population-projections/2012-based-extra-variants/rft-table-z8-zipped-population-data-files---england.zip

Thus we calculate that for the above example, a reduction in CHD deaths of 6 per 100,000 for Men 65-74 in IMDQ5 in the year 2015 would represent 22 CHD DPP in absolute numbers (6 per 100,000 x 365,000).

The CHD DPP for the year 2020 were translated to Life Years Gained using the data in the table below. We assumed that 26% of CHD DPP were among those with the disease state “diagnosed CHD”, another 26% with “undiagnosed CHD”, and the remaining 48% with “no CHD”. From the example above, 22 CHD DPP for Men 65-74 in IMDQ5 would result in an average of 8.4 LYG per DPP (0.26 x 3.6 + 0.26 x 7.6 + 0.48 x 11.5), or in total 185 LYG (8.4 LYG per DPP x 22 DPP). As described in the main text (Table 1), the QALY gained would be 155 QALY (185 LYG x 0.84 QALY per LYG).

We repeated this process for all age groups and IMDQs, for both genders. There are a small number of CHD incidence cases among the age group 25-34, but the number of annual deaths is in the low single digits. The average annual DPP would therefore be less than zero at the scale of reduction we model and not meaningful to include. We did retain this age group in the cost savings portion of the analysis because the reduction in incidence could be non-zero, though still very small.

Table E: Median survival (years) by Age, Gender, and IMDQ for each of three CHD disease states. The proportion of CHD deaths in each was assumed to be 26% (diagnosed CHD), 26% (undiagnosed CHD), and 48% (no CHD). These percentages were based on the contribution of treatments to the overall decline in CHD mortality in the IMPACT-SEC model (Bajekal et al. 2012).

**CHD Disease State**	**Gender**	**Age**	**IMDQ5**	**IMDQ4**	**IMDQ3**	**IMDQ2**	**IMDQ1**
CHD, Diagnosed	M	35-44	23.7	24.6	25.2	25.5	25.9
CHD, Diagnosed	M	45-54	11.8	12.3	12.6	12.8	12.9
CHD, Diagnosed	M	55-64	6.4	6.7	6.8	6.9	7.0
CHD, Diagnosed	M	65-74	3.6	3.8	3.8	3.9	4.0
CHD, Diagnosed	M	75-84	2.4	2.5	2.5	2.6	2.6
CHD, Diagnosed	M	85+	1.9	1.9	1.9	1.9	1.9
CHD, Diagnosed	F	35-44	23.7	24.6	25.2	25.5	25.9
CHD, Diagnosed	F	45-54	11.8	12.3	12.6	12.8	12.9
CHD, Diagnosed	F	55-64	6.4	6.7	6.8	6.9	7.0
CHD, Diagnosed	F	65-74	3.6	3.8	3.8	3.9	4.0
CHD, Diagnosed	F	75-84	2.4	2.5	2.5	2.6	2.6
CHD, Diagnosed	F	85+	1.8	1.8	1.9	1.9	1.9
CHD, Undiagnosed	M	35-44	29.3	30.4	31.1	31.6	32.0
CHD, Undiagnosed	M	45-54	19.0	19.8	20.2	20.5	20.8
CHD, Undiagnosed	M	55-64	12.3	12.8	13.1	13.3	13.5
CHD, Undiagnosed	M	65-74	7.6	7.8	8.0	8.1	8.3
CHD, Undiagnosed	M	75-84	4.4	4.6	4.7	4.7	4.8
CHD, Undiagnosed	M	85+	2.7	2.8	2.9	2.9	3.0
CHD, Undiagnosed	F	35-44	35.9	37.3	38.1	38.7	39.2
CHD, Undiagnosed	F	45-54	31.3	32.5	33.3	33.8	34.2
CHD, Undiagnosed	F	55-64	26.9	27.9	28.6	29.0	29.4
CHD, Undiagnosed	F	65-74	16.7	17.4	17.8	18.0	18.3
CHD, Undiagnosed	F	75-84	10.3	10.7	10.9	11.1	11.2
CHD, Undiagnosed	F	85+	5.9	6.1	6.3	6.4	6.5
NO CHD	M	35-44	34.9	36.3	37.1	37.6	38.2
NO CHD	M	45-54	26.3	27.3	27.9	28.3	28.7
NO CHD	M	55-64	18.3	19.0	19.4	19.7	20.0
NO CHD	M	65-74	11.5	11.9	12.2	12.4	12.6
NO CHD	M	75-84	6.4	6.6	6.8	6.9	7.0
NO CHD	M	85+	3.7	3.8	3.9	4.0	4.0
NO CHD	F	35-44	39.0	40.5	41.4	42.0	42.6
NO CHD	F	45-54	30.1	31.3	32.0	32.5	32.9
NO CHD	F	55-64	21.6	22.5	23.0	23.3	23.7
NO CHD	F	65-74	14.1	14.7	15.0	15.2	15.4
NO CHD	F	75-84	8.2	8.5	8.7	8.8	9.0
NO CHD	F	85+	4.4	4.6	4.7	4.8	4.8

Inequality in Health Outcomes

The Slope Index of Inequality [33] is a measure of absolute inequality. In our policy model, we are interested in the change in absolute inequality, by comparing the Slope Index under the "do-nothing" scenario of past trends with the Slope Index with each policy option implemented. An example of the calculation follows based on the following data:

IMDQ	Deaths (Baseline)	Reduction	Deaths (With Policy)
1	10000	600	9400
2	12000	1000	11000
3	14000	1200	12800
4	15000	1200	13800
5	21000	3000	18000

The number of CHD deaths at baseline are for the "do-nothing" scenario based on past trends. The reduction is for a hypothetical policy option that reduces TFA consumption. The number of deaths with the policy implemented is the difference between the baseline and the reduction. This can be illustrated more easily on a plot:

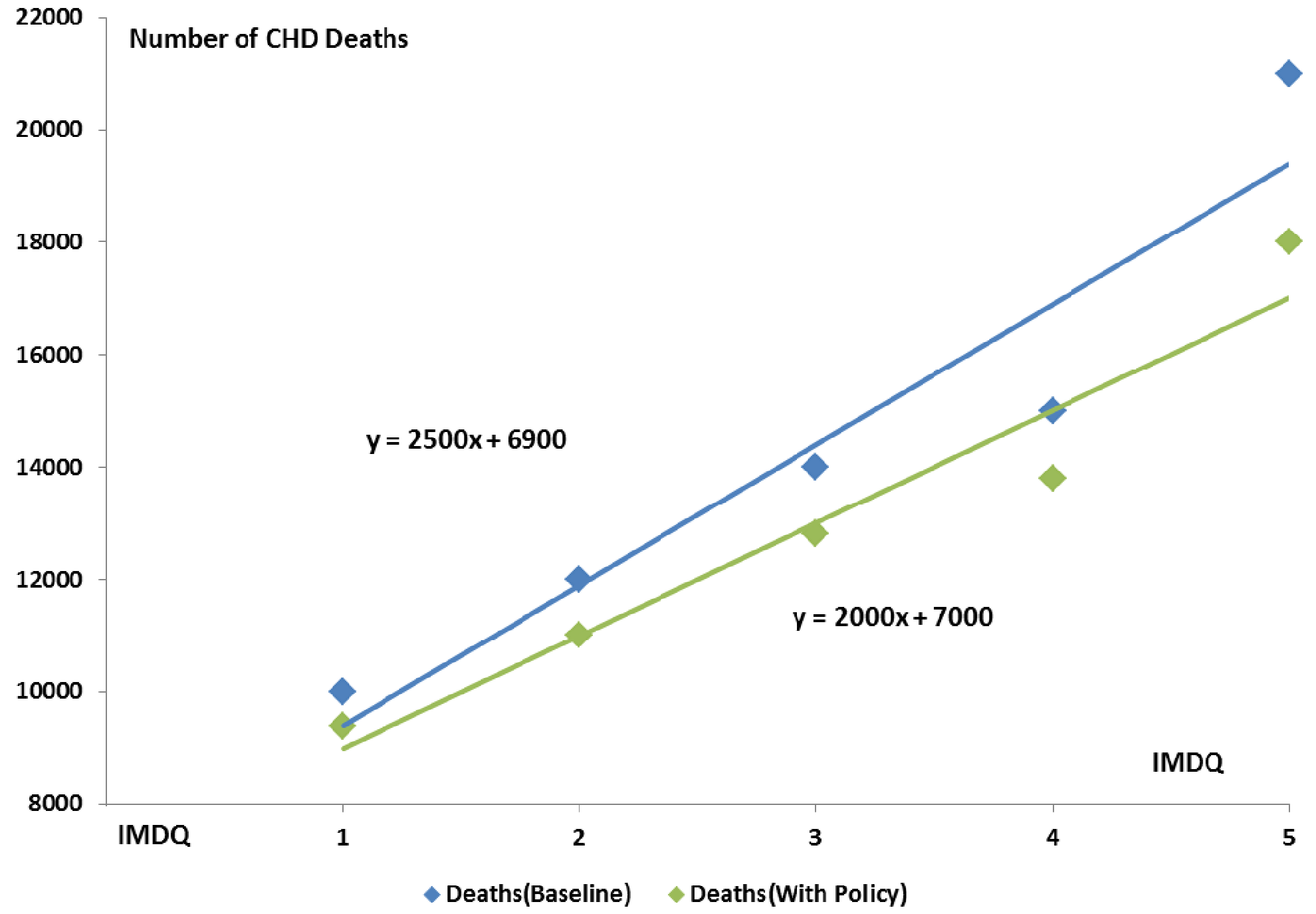

Figure A: Illustration of Slope Index calculation.

First, the number of CHD deaths is plotted on the vertical axis with the IMDQ on the horizontal axis. Then for each scenario (baseline and with policy), a simple linear regression is fitted. The Slope Index is the *fitted* difference between IMDQ5 and IMDQ1. This is simply the slope of the line multiplied by four because 5 minus 1 is 4. For the "baseline" example, the Slope Index is 10,000 (4 times 2,500), while for the scenario "with policy" the Slope Index is 8,000 (4 times 2,000). The *change* in slope index is thus a reduction of 2,000 (8,000 minus 10,000 = -2,000).

SUPPLEMENTARY RESULTS

First we provide the CHD DPP for each age group and gender. In some cases, the numbers are listed as simply less than 10 ("*<10*") to avoid giving the impression of over-precision.

Table F: Total CHD deaths for 2015-2020 and reductions associated with each policy option, stratified by IMDQ and Gender, for the Ages 35-44.

MEN AGES 35-44

DEATHS	2015-2020					
	BASELINE	REDUCTIONS				
IMDQ		BAN	LABEL (NO SEC Gradient)	FAST FOOD	LABEL (SEC Gradient)	RESTAURANT
1	160	*< 10*	*< 10*	*< 10*	*< 10*	*< 10*
2	220	*< 10*	*< 10*	*< 10*	*< 10*	*< 10*
3	280	13(10 to 20)	*< 10*	*< 10*	*< 10*	*< 10*
4	470	40(20 to 80)	20(10 to 40)	10(10 to 30)	10(0 to 20)	10(0 to 20)
5	680	80(40 to 150)	40(20 to 70)	30(20 to 60)	20(10 to 40)	20(10 to 30)
	1810	150(60 to 260)	70(30 to 130)	60(20 to 100)	40(20 to 80)	40(20 to 70)

WOMEN AGES 35-44

DEATHS	2015-2020					
	BASELINE	REDUCTIONS				
IMDQ		BAN	LABEL (NO SEC Gradient)	FAST FOOD	LABEL (SEC Gradient)	RESTAURANT
1	40	*< 10*	*< 10*	*< 10*	*< 10*	*< 10*
2	40	*< 10*	*< 10*	*< 10*	*< 10*	*< 10*
3	90	*< 10*	*< 10*	*< 10*	*< 10*	*< 10*
4	110	*< 10*	*< 10*	*< 10*	*< 10*	*< 10*
5	200	20(10 to 50)	10(0 to 30)	10(0 to 20)	10(0 to 10)	10(0 to 10)
		40(20 to 90)	20(10 to 50)	20(10 to 30)	10(0 to 30)	10(0 to 20)

Table G: Total CHD deaths for 2015-2020 and reductions associated with each policy option, stratified by IMDQ and Gender, for the Ages 45-54.

MEN AGES 45-54

DEATHS	2015-2020					
	BASELINE	REDUCTIONS				
IMDQ		BAN	LABEL (NO SEC Gradient)	FAST FOOD	LABEL (SEC Gradient)	RESTAURANT
1	790	10(0 to 20)	*< 10*	*< 10*	*< 10*	*< 10*
2	1030	20(10 to 40)	10(0 to 20)	10(0 to 10)	10(0 to 20)	10(0 to 10)
3	1300	40(20 to 70)	20(10 to 40)	10(10 to 20)	20(10 to 30)	10(10 to 20)
4	1870	110(50 to 200)	60(20 to 100)	40(20 to 70)	40(20 to 60)	30(10 to 50)
5	2760	240(110 to 400)	120(50 to 200)	100(50 to 170)	60(30 to 100)	60(20 to 100)
		430(190 to 730)	210(90 to 370)	160(80 to 280)	130(60 to 220)	110(50 to 190)

WOMEN AGES 45-54

DEATHS	2015-2020					
	BASELINE	REDUCTIONS				
IMDQ		BAN	LABEL (NO SEC Gradient)	FAST FOOD	LABEL (SEC Gradient)	RESTAURANT
1	180	*< 10*	*< 10*	*< 10*	*< 10*	*< 10*
2	250	*< 10*	*< 10*	*< 10*	*< 10*	*< 10*
3	350	12(5 to 20)	10(0 to 10)	*< 10*	*< 10*	*< 10*
4	480	30(10 to 50)	10(10 to 30)	10(0 to 20)	10(0 to 20)	10(0 to 10)
5	800	70(30 to 130)	30(10 to 60)	30(10 to 50)	20(10 to 30)	20(10 to 30)
		120(50 to 220)	60(20 to 110)	40(20 to 80)	30(10 to 60)	30(10 to 60)

Table H: Total CHD deaths for 2015-2020 and reductions associated with each policy option, stratified by IMDQ and Gender, for the Ages 55-64.

MEN AGES 55-64

DEATHS	2015-2020					
	BASELINE	REDUCTIONS				
IMDQ		BAN	LABEL (NO SEC Gradient)	FAST FOOD	LABEL (SEC Gradient)	RESTAURANT
1	2030	20(10 to 30)	10(0 to 10)	*< 10*	10(0 to 10)	10(0 to 10)
2	2520	40(20 to 70)	20(10 to 30)	10(10 to 20)	20(10 to 30)	10(10 to 20)
3	3170	80(30 to 130)	40(20 to 60)	20(10 to 40)	30(10 to 50)	30(10 to 40)
4	3810	170(70 to 290)	80(30 to 140)	60(30 to 100)	50(20 to 90)	40(20 to 80)
5	5450	350(150 to 570)	170(70 to 290)	140(70 to 240)	90(40 to 140)	80(30 to 130)
		650(290 to 1090)	320(140 to 540)	240(110 to 400)	190(80 to 320)	170(70 to 290)

WOMEN AGES 55-64

DEATHS	2015-2020					
	BASELINE	REDUCTIONS				
IMDQ		BAN	LABEL (NO SEC Gradient)	FAST FOOD	LABEL (SEC Gradient)	RESTAURANT
1	640	*< 10*	*< 10*	*< 10*	*< 10*	*< 10*
2	840	14(10 to 20)	*< 10*	*< 10*	*< 10*	*< 10*
3	1040	30(10 to 40)	10(10 to 20)	10(0 to 10)	10(0 to 20)	10(0 to 10)
4	1240	50(20 to 100)	30(10 to 50)	20(10 to 30)	20(10 to 30)	10(10 to 30)
5	1790	110(50 to 200)	60(20 to 100)	50(20 to 80)	30(10 to 50)	30(10 to 50)
		210(90 to 380)	100(40 to 190)	80(40 to 140)	60(30 to 110)	60(20 to 100)

Table I: Total CHD deaths for 2015-2020 and reductions associated with each policy option, stratified by IMDQ and Gender, for the Ages 65-74.

MEN AGES 65-74

DEATHS	2015-2020					
	BASELINE	REDUCTIONS				
IMDQ		BAN	LABEL (NO SEC Gradient)	FAST FOOD	LABEL (SEC Gradient)	RESTAURANT
1	4670	30(10 to 50)	10(10 to 20)	10(0 to 10)	10(10 to 20)	10(0 to 20)
2	5580	70(30 to 110)	30(10 to 60)	20(10 to 30)	30(10 to 50)	20(10 to 40)
3	6870	130(60 to 210)	60(30 to 100)	40(20 to 70)	50(20 to 80)	40(20 to 70)
4	7540	250(110 to 430)	120(50 to 210)	90(40 to 150)	80(30 to 130)	70(30 to 110)
5	9880	470(210 to 790)	230(100 to 390)	190(90 to 320)	120(50 to 200)	110(50 to 180)
		940(420 to 1590)	460(200 to 790)	340(160 to 580)	280(120 to 480)	250(110 to 420)

WOMEN AGES 65-74

DEATHS	2015-2020					
	BASELINE	REDUCTIONS				
IMDQ		BAN	LABEL (NO SEC Gradient)	FAST FOOD	LABEL (SEC Gradient)	RESTAURANT
1	1790	10(0 to 20)	10(0 to 10)	*< 10*	10(0 to 10)	*< 10*
2	2260	30(10 to 50)	10(10 to 20)	10(0 to 10)	10(0 to 20)	10(0 to 20)
3	2730	50(20 to 90)	20(10 to 40)	20(10 to 30)	20(10 to 30)	20(10 to 30)
4	3340	110(50 to 200)	50(20 to 100)	40(20 to 70)	30(10 to 60)	30(10 to 50)
5	4160	200(90 to 360)	100(40 to 180)	80(40 to 150)	50(20 to 90)	40(20 to 80)
		390(170 to 710)	190(80 to 350)	140(70 to 260)	120(50 to 210)	100(40 to 190)

Table J: Total CHD deaths for 2015-2020 and reductions associated with each policy option, stratified by IMDQ and Gender, for the Ages 75-84.

MEN AGES 75-84

DEATHS	2015-2020					
	BASELINE	REDUCTIONS				
IMDQ		BAN	LABEL (NO SEC Gradient)	FAST FOOD	LABEL (SEC Gradient)	RESTAURANT
1	8430	50(20 to 80)	20(10 to 40)	10(10 to 20)	20(10 to 40)	20(10 to 30)
2	9430	110(50 to 180)	50(20 to 90)	30(10 to 50)	50(20 to 80)	40(20 to 60)
3	10880	190(80 to 310)	90(40 to 150)	60(30 to 100)	70(30 to 110)	60(30 to 100)
4	10850	340(150 to 590)	160(70 to 290)	120(50 to 200)	100(40 to 180)	90(40 to 160)
5	12070	550(240 to 910)	270(120 to 450)	220(100 to 370)	140(60 to 230)	120(50 to 210)
		1230(540 to 2080)	600(260 to 1020)	440(200 to 740)	380(160 to 640)	330(140 to 560)

WOMEN AGES 75-84

DEATHS	2015-2020					
	BASELINE	REDUCTIONS				
IMDQ		BAN	LABEL (NO SEC Gradient)	FAST FOOD	LABEL (SEC Gradient)	RESTAURANT
1	4840	30(10 to 50)	10(10 to 20)	10(0 to 10)	10(0 to 10)	10(0 to 20)
2	5690	60(30 to 120)	30(10 to 60)	20(10 to 30)	10(0 to 20)	20(10 to 40)
3	6530	110(50 to 190)	50(20 to 90)	40(20 to 60)	20(10 to 30)	40(20 to 60)
4	7130	220(100 to 410)	110(40 to 200)	80(30 to 140)	30(10 to 60)	60(20 to 110)
5	7650	340(150 to 620)	170(70 to 310)	140(60 to 250)	50(20 to 90)	80(30 to 140)
		770(340 to 1390)	370(150 to 680)	270(130 to 490)	240(100 to 420)	210(90 to 370)

Table K: Total CHD deaths for 2015-2020 and reductions associated with each policy option, stratified by IMDQ and Gender, for the Ages 85+.

MEN AGES 85+

DEATHS	2015-2020					
	BASELINE	REDUCTIONS				
IMDQ		BAN	LABEL (NO SEC Gradient)	FAST FOOD	LABEL (SEC Gradient)	RESTAURANT
1	9650	50(20 to 90)	30(10 to 40)	10(10 to 20)	30(10 to 40)	20(10 to 40)
2	10240	120(50 to 190)	60(20 to 100)	30(10 to 50)	50(20 to 80)	40(20 to 70)
3	10910	180(80 to 310)	90(40 to 150)	60(30 to 100)	70(30 to 110)	60(30 to 100)
4	9770	300(130 to 520)	150(60 to 260)	100(50 to 180)	90(40 to 160)	80(30 to 140)
5	8580	380(170 to 630)	180(80 to 310)	150(70 to 260)	90(40 to 160)	90(40 to 150)
		1030(460 to 1750)	500(220 to 860)	360(170 to 610)	330(140 to 560)	290(120 to 490)

WOMEN AGES 85+

DEATHS	2015-2020					
	BASELINE	REDUCTIONS				
IMDQ		BAN	LABEL (NO SEC Gradient)	FAST FOOD	LABEL (SEC Gradient)	RESTAURANT
1	10170	60(30 to 100)	30(10 to 50)	10(10 to 20)	10(10 to 20)	20(10 to 40)
2	11680	130(60 to 230)	60(30 to 110)	30(20 to 60)	30(10 to 50)	50(20 to 80)
3	12440	210(90 to 360)	100(40 to 180)	70(30 to 120)	40(20 to 70)	70(30 to 120)
4	12090	370(160 to 680)	180(70 to 330)	130(60 to 230)	70(30 to 120)	100(40 to 180)
5	10190	450(200 to 810)	220(90 to 400)	180(80 to 330)	90(30 to 150)	100(40 to 180)
		1210(530 to 2180)	590(240 to 1070)	420(190 to 760)	390(160 to 680)	340(140 to 590)

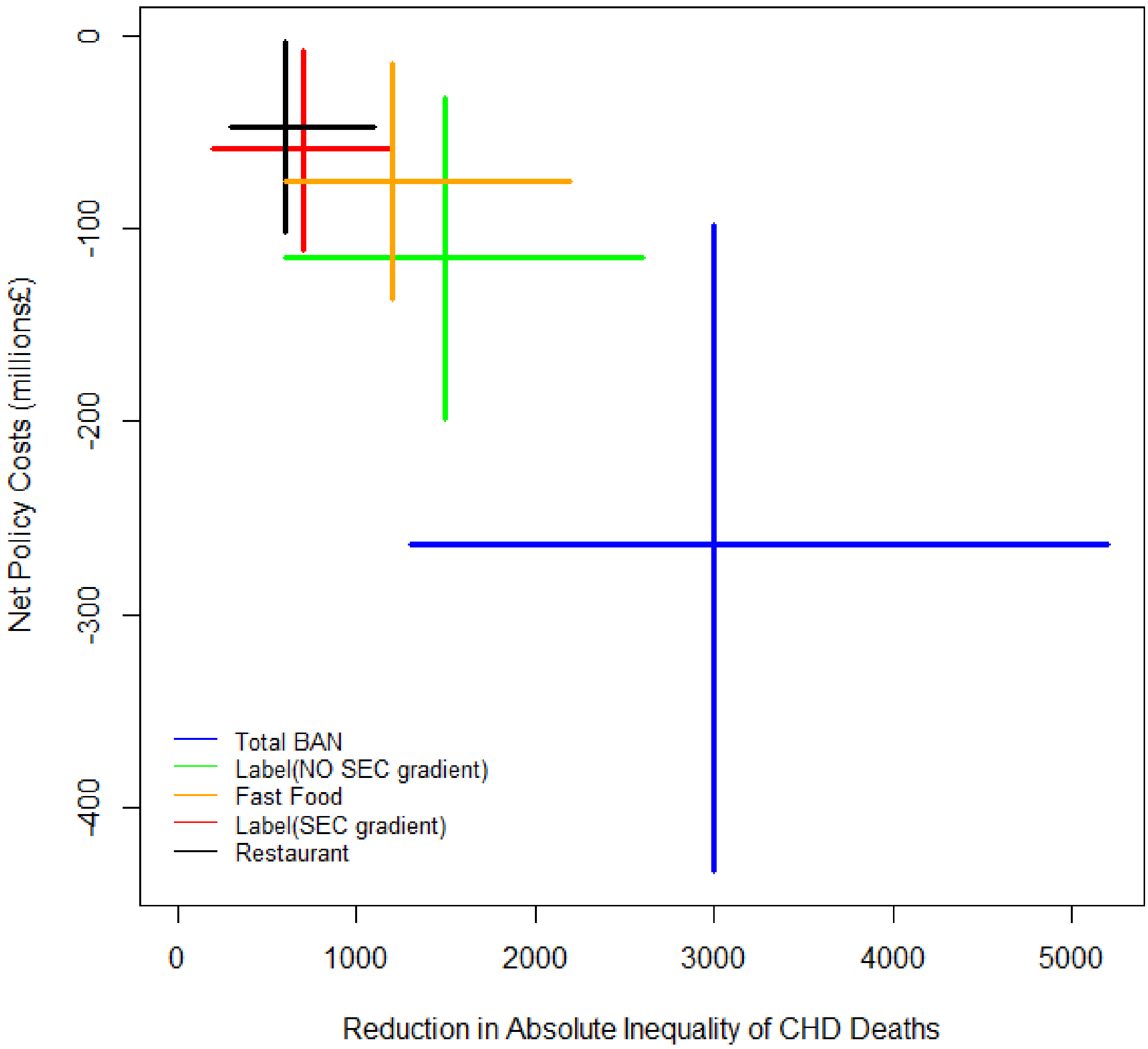

Figure B: Net costs (negative is saving) vs. change in inequality of CHD mortality (negative is reduction). Net costs are from the optimistic scenario (Table 7) where industry reformulation costs are included in part of the normal product life cycle and thus zero. Compared to Figure 2 in the main paper, here we see less overlap in the cost estimates.

Next we present a one-way sensitivity analysis of the TFA consumption gradient, where we assume that each IMDQ has the same average TFA consumption. Table L shows the TFA consumption and reductions modelled for each policy under this scenario (compare to Table 3 in main text).

Table M translates the reductions in TFA consumption to CHD DPP for each policy option for each IMDQ (compare to Table 4 in main text). For each policy option, the percentage reductions in CHD deaths would vary little by IMDQ (moving down in a column), with the slight differences being only due to different age-gender distributions within each IMDQ. The net effect would be about one-third lower compared to the main analysis, but the absolute inequality reduction would be substantially lower. The restaurant scenario and the labelling scenario that favour IMDQ1 would be essentially neutral at reducing absolute inequality. The policies that benefit all IMDQs equally (total ban, labelling without a gradient) or that favour IMDQ5 (fast food) could still reduce absolute inequality of CHD mortality, but the effect would be small.

Next we show the cost savings associated with each policy option under the assumption of constant TFA consumption (Table N, equivalent to Table 6 in main text). The cost savings would be lower by about the same percentage as the CHD DPP (Table M), i.e. a little more than one-third lower.

Table L: Baseline TFA consumption (%E) and reductions associated with modelled policy options, if we assume that TFA consumption does not vary by IMDQ. The maximum reduction is down to 0.4%E based on ruminant TFA remaining in the diet. Compare with Table 3 in main text.

TRANS FAT CONSUMPTION (%ENERGY)

IMDQ	TOTAL CONSUMPTION	RUMINANT SOURCES	REDUCTIONS TOTAL BAN	LABEL (NO SEC Gradient)	FAST FOOD	LABEL (SEC gradient)	RESTAURANT
1	0.70 (0.66 to 0.74)	0.40	0.30 (0.26 to 0.34)	0.15 (0.13 to 0.17)	0.07 (0.06 to 0.08)	0.15 (0.14 to 0.16)	0.12 (0.10 to 0.14)
2	0.70 (0.66 to 0.74)	0.40	0.30 (0.26 to 0.34)	0.15 (0.13 to 0.17)	0.08 (0.07 to 0.09)	0.13 (0.12 to 0.14)	0.10 (0.08 to 0.12)
3	0.70 (0.66 to 0.74)	0.40	0.30 (0.26 to 0.34)	0.15 (0.13 to 0.17)	0.10 (0.09 to 0.11)	0.11 (0.10 to 0.12)	0.10 (0.09 to 0.11)
4	0.70 (0.66 to 0.74)	0.40	0.30 (0.26 to 0.34)	0.15 (0.13 to 0.17)	0.10 (0.08 to 0.12)	0.09 (0.07 to 0.11)	0.08 (0.07 to 0.09)
5	0.70 (0.66 to 0.74)	0.40	0.30 (0.26 to 0.34)	0.15 (0.13 to 0.17)	0.12 (0.10 to 0.14)	0.07 (0.05 to 0.09)	0.07 (0.06 to 0.08)
AVERAGE	0.70 (0.66 to 0.74)	0.40	0.30 (0.26 to 0.34)	0.15 (0.13 to 0.17)	0.09 (0.07 to 0.11)	0.11 (0.09 to 0.13)	0.09 (0.08 to 0.10)

TOTAL CONSUMPTION	Estimated from dietary surveys [15,16]
RUMINANT SOURCES	TFA consumption from ruminant sources [15,16]
TOTAL BAN	Eliminate all TFA from processed foods, i.e. Reduce to 0.4%
LABEL(NO SEC gradient)	Reduce by 49% of BAN amount for all IMDQ [23]
LABEL(SEC gradient)	IMDQ1 has max label effect (49%), linear down to IMDQ5 at 50% [24] of that (50% of 49% = 24.5%)
RESTAURANT	TFA consumption away from home proportional to food spending away from home (favours IMDQ1)
FAST FOOD	Reverse the IMDQ gradient of expenditure away from home (favours IMDQ5)

Table M: Total CHD deaths for 2015-2020 and reductions associated with each policy option, stratified by IMDQ [% reductions in brackets], if TFA consumption does not vary across IMDQ. Number in parenthesis are 95% confidence intervals from probabilistic sensitivity analysis. Absolute inequality in CHD mortality across IMDQs is measured by the Slope Index [28]. We report the baseline value and the reduction (e.g. a reduction of 400 means the Slope Index of a total ban would be 20,000). Compare with Table 4 in main text.

DEATHS	2015-2020					
	BASELINE	REDUCTIONS				
		BAN	LABEL	FAST FOOD	LABEL	RESTAURANT
IMDQ			(NO SEC Gradient)		(SEC Gradient)	
1	43000	800 [1.9%]	400 [0.9%]	200 [0.5%]	400 [0.9%]	300 [0.7%]
		(300 to 1300)	(200 to 600)	(100 to 300)	(200 to 700)	(100 to 500)
2	50000	900 [1.8%]	400 [0.8%]	200 [0.4%]	400 [0.8%]	300 [0.6%]
		(400 to 1600)	(200 to 800)	(100 to 400)	(200 to 700)	(100 to 500)
3	57000	1000 [1.8%]	500 [0.9%]	300 [0.5%]	400 [0.7%]	300 [0.5%]
		(400 to 1800)	(200 to 900)	(100 to 600)	(200 to 700)	(100 to 600)
4	59000	1100 [1.9%]	500 [0.8%]	400 [0.7%]	300 [0.5%]	300 [0.5%]
		(400 to 2000)	(200 to 1000)	(200 to 700)	(100 to 600)	(100 to 500)
5	64000	1200 [1.9%]	600 [0.9%]	500 [0.8%]	300 [0.5%]	300 [0.5%]
		(500 to 2200)	(300 to 1100)	(200 to 900)	(100 to 500)	(100 to 500)
TOTAL	273000	5000 [1.8%]	2400 [0.9%]	1600 [0.6%]	1800 [0.7%]	1500 [0.5%]
		(2000 to 8900)	(1100 to 4400)	(700 to 2900)	(800 to 3200)	(500 to 2600)
ABSOLUTE	20400	400 [2.0%]	200 [1.0%]	300 [1.5%]	-100 [-0.5%]	0 [0.0%]
INEQUALITY		(200 to 900)	(100 to 500)	(100 to 600)	(-100 to -200)	(0 to 0)

Table N: Direct health care savings, averted productivity loss and informal care savings for 2015-2020 with each policy option, assuming TFA consumption does not vary by IMDQ. Compare to Table 6 in main text.

HEALTHCARE SAVINGS (2015-2020)

IMDQ		BAN	LABEL (NO SEC Gradient)	FAST FOOD	LABEL (SEC Gradient)	RESTAURANT
	1	4.7 (2.0 to 8.1)	2.3 (1.0 to 4.0)	1.1 (0.5 to 1.8)	2.3 (1.0 to 4.0)	1.9 (0.8 to 3.3)
	2	5.5 (2.3 to 9.8)	2.7 (1.1 to 4.8)	1.5 (0.6 to 2.6)	2.4 (1.0 to 4.2)	1.9 (0.8 to 3.4)
	3	5.9 (2.5 to 10.4)	2.9 (1.2 to 5.1)	1.9 (0.8 to 3.4)	2.2 (0.9 to 3.9)	1.9 (0.8 to 3.4)
	4	6.4 (2.6 to 11.8)	3.2 (1.3 to 5.8)	2.2 (0.9 to 4.1)	2.0 (0.8 to 3.7)	1.7 (0.7 to 3.1)
	5	7.3 (3.1 to 13.2)	3.6 (1.5 to 6.5)	3.0 (1.3 to 5.3)	1.8 (0.8 to 3.3)	1.7 (0.7 to 3.0)
TOTAL		29.8 (12.6 to 53.4)	14.7 (6.2 to 26.3)	9.6 (4.1 to 17.2)	10.7 (4.5 to 19.1)	9.1 (3.8 to 16.2)

AVERTED PRODUCTIVITY LOSS (2015-2020)

IMDQ		BAN	LABEL (NO SEC Gradient)	FAST FOOD	LABEL (SEC Gradient)	RESTAURANT
	1	5.3 (2.3 to 9.2)	2.6 (1.1 to 4.5)	1.2 (0.5 to 2.1)	2.6 (1.1 to 4.5)	2.1 (0.9 to 3.7)
	2	6.4 (2.7 to 11.5)	3.2 (1.3 to 5.7)	1.7 (0.7 to 3.1)	2.8 (1.2 to 5.0)	2.2 (0.9 to 4.0)
	3	7.4 (3.1 to 13.0)	3.7 (1.6 to 6.4)	2.4 (1.0 to 4.2)	2.7 (1.2 to 4.8)	2.4 (1.0 to 4.2)
	4	8.8 (3.6 to 16.2)	4.3 (1.8 to 8.0)	3.0 (1.3 to 5.6)	2.7 (1.1 to 5.0)	2.3 (1.0 to 4.3)
	5	11.5 (4.8 to 20.6)	5.7 (2.4 to 10.2)	4.7 (2.0 to 8.4)	2.8 (1.2 to 5.1)	2.6 (1.1 to 4.7)
TOTAL		39.3 (16.6 to 70.4)	19.4 (8.2 to 34.8)	13.0 (5.5 to 23.3)	13.7 (5.8 to 24.5)	11.7 (4.9 to 21.0)

INFORMAL CARE SAVINGS (2015-2020)

IMDQ		BAN	LABEL (NO SEC Gradient)	FAST FOOD	LABEL (SEC Gradient)	RESTAURANT
	1	21.7 (9.3 to 37.7)	10.7 (4.6 to 18.6)	4.9 (2.1 to 8.5)	10.7 (4.6 to 18.6)	8.8 (3.7 to 15.3)
	2	25.4 (10.7 to 45.6)	12.5 (5.3 to 22.5)	6.8 (2.8 to 12.1)	11.0 (4.6 to 19.7)	8.8 (3.7 to 15.8)
	3	27.5 (11.7 to 48.6)	13.6 (5.8 to 24.0)	8.9 (3.8 to 15.7)	10.2 (4.3 to 18.0)	8.9 (3.8 to 15.7)
	4	29.9 (12.3 to 55.2)	14.7 (6.1 to 27.2)	10.4 (4.3 to 19.1)	9.2 (3.8 to 17.0)	8.0 (3.3 to 14.7)
	5	34.4 (14.5 to 61.7)	17.0 (7.1 to 30.4)	13.9 (5.9 to 25.0)	8.5 (3.6 to 15.3)	7.8 (3.3 to 13.9)
TOTAL		138.9 (58.5 to 248.8)	68.5 (28.9 to 122.7)	44.9 (18.9 to 80.4)	49.6 (20.9 to 88.8)	42.2 (17.8 to 75.6)
GRAND TOTAL		208.0 (87.6 to 372.6)	102.6 (43.2 to 183.8)	67.5 (28.4 to 120.9)	73.9 (31.1 to 132.4)	63.0 (26.5 to 112.9)

Finally in Table O, we show the costs to industry and to government, combined with the total cost savings above. Here it seems likely that the policies would not be strictly cost-saving from a societal perspective. In fact, because the TFA reduction would be more constant across IMDQ (compared to the main analysis) and proportional to any reformulation costs, the different policy options have nearly identical net costs. We stress that reformulation costs to industry are a worst-case scenario because many products could likely be reformulated as part the natural cycle within a timeframe that complied any policy implementation. If we focus on the three costs that are likely to be shared by any policy (legislation, monitoring, on-going expenses), any policy option would still be cost-saving and dominant over a scenario of "do-nothing" where TFA consumption remains constant.

Table O: Policy costs to government for legislation (one-time) and monitoring (annually). Costs to industry associated with possible lost profits (annually) and reformulation (one-time). If reformulation occurs as part of normal product life-cycle, there would be no incremental cost for reformulation. Units are Millions £. Analogous to Table 7 in main text.

		COMMON TO ALL POLICIES	Industry costs (Worst-Case)				
			BAN	LABEL (NO SEC gradient)	FAST FOOD	LABEL (SEC gradient)	RESTAURANT
GOV'T	Legislation	5.0					
	Monitoring	16.6					
INDUSTRY	On-going Expenses	11.0					
	Reformulation		200.0	94.5	55.1	66.4	55.1
SUM ALL COSTS		32.6	232.6	127.1	87.7	99.0	87.7
SUM ALL SAVINGS (TABLE S14)			208.0	102.6	73.9	63.0	67.5
			(87.6 to 372.6)	(43.2 to 183.8)	(31.1 to 132.4)	(26.5 to 112.9)	(28.4 to 120.9)
OPTIMISTIC NET COST			-175.4	-70.0	-34.9	-41.3	-30.4
			(-340.0 to -55.0)	(-151.2 to -10.6)	(-88.3 to 4.2)	(-99.8 to 1.5)	(-80.2 to 6.1)
PESSIMISTIC NET COST			24.6	24.5	13.8	36.0	20.2
			(-140.0 to 145.0)	(-56.7 to 83.9)	(-44.7 to 56.6)	(-13.9 to 72.4)	(-33.2 to 59.3)
